# ^18^F-FDG PET/CT Findings in a Patient with Chikungunya Virus Infection

**DOI:** 10.3390/diagnostics7030049

**Published:** 2017-08-25

**Authors:** Michala Vaaben Rose, Anna Sophie L. Kjaer, Elena Markova, Jesper Graff

**Affiliations:** 1 Department of Infectious Diseases, Copenhagen University Hospital Hvidovre, Kettegaard Allé 30, Hvidovre DK-2650, Denmark; 2 Department of Infectious Diseases, Copenhagen University Hospital Hvidovre, Kettegaard Allé 30, Hvidovre DK-2650, Denmark; vqs488@alumni.ku.dk; 3 Department of Radiology, Copenhagen University Hospital Hvidovre, Kettegaard Allé 30, Hvidovre DK-2650, Denmark; elena.markova.01@regionh.dk; 4 Department of Clinical Physiology & Nuclear Medicine, Copenhagen University Hospital Hvidovre, Kettegaard Allé 30, Hvidovre DK-2650, Denmark; jesper.graff@regionh.dk

**Keywords:** ^18^F-FDG PET/CT, Chikungunya virus, infection imaging

## Abstract

We present a case demonstrating the diagnostic work-up and follow-up of a patient with Chikungunya infection. An ^18^F-FDG PET/CT performed four weeks after debut of symptoms revealed pathological ^18^F-FDG uptake in enlarged lymph nodes on both side of the diaphragm, and inflammation of both shoulder and hip joints. Lymphoma and infection were the main differential diagnoses. Follow-up ^18^F-FDG PET/CT scan in the patient performed 14 weeks after the abnormal scan, revealed almost complete resolution of the metabolically active disease. This case is to our knowledge the first to demonstrate sequential ^18^F-FDG PET/CT scan results in a patient with Chikungunya virus infection.

**Figure 1:**
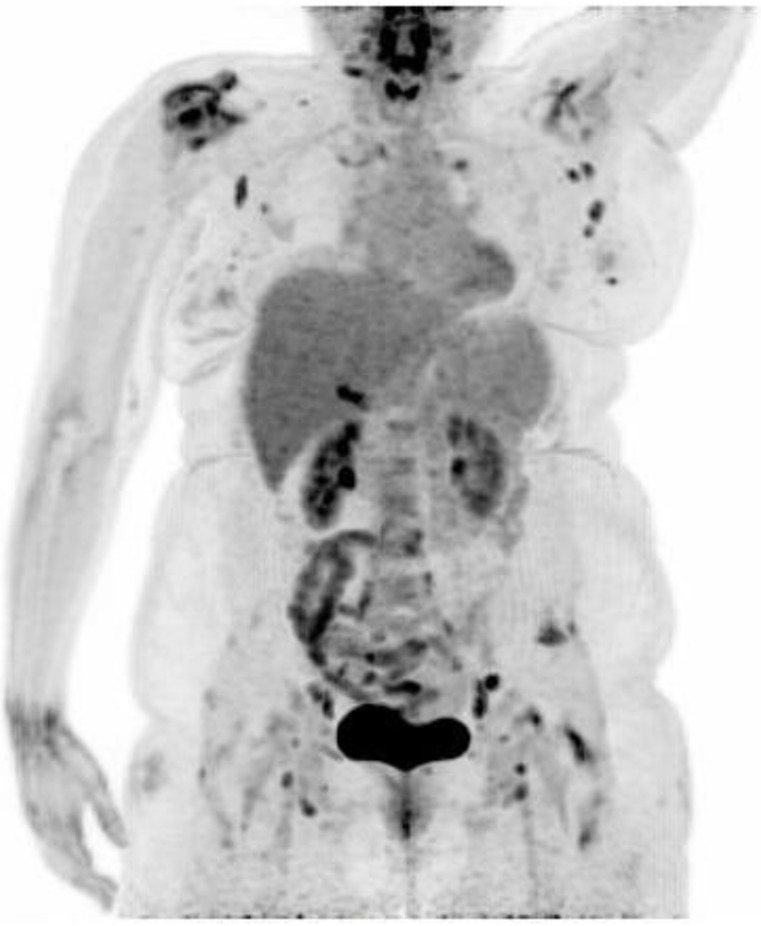
A 50-year old woman of Indian descent living in Denmark, with a prior history of knee arthrosis, presented with fever, myalgia and arthralgia which had developed during a return visit to New Delhi, India. Two weeks after returning to Denmark the generalised arthralgia and myalgia persisted to a degree where the patient was mainly confined to her bed. The symptoms were accompanied by extreme fatigue and fever sensation. When examined, the patient’s upper extremities were almost immobile due to soreness of shoulder, elbow and wrist joints, as well as reduced mobility of lower extremities, with slight edema of both feet. On inspection there were no visible signs of joint inflammation or skin rash. On admission we found elevated a sedimentation rate of 51 mm (normal range: 2–20 mm), C-reactive protein 25 mg/L (normal range: 0–10 mg/L), d-dimer 1, 9 FEU (fibrinogen equivalent units)/L (normal range < 0.5 FEU/L) and slightly elevated Lactate dehydrogenase at 212 U/L (normal range: 105–205 U/L). White blood cell count, creatinin kinase, antineutrophil cytoplasmic antibody, and antinuclear antibody were in the normal range. Dengue IgG and IgM were negative. NSAID treatment in combination with tramadole was initiated with no effect on the patient’s severe arthralgia. An ^18^F-FDG PET/CT (fluorine-18 fluoro-2-deoxy-d-glucose positron emission tomography/computed tomography) was performed four weeks after debut of symptoms. Due to severe pain the patient was not able to place her right arm over her head during the scanning. The scan revealed pathological ^18^F-FDG uptake in enlarged lymph nodes on both side of the diaphragm, inflammation of both shoulder and hip joints, as well as ^18^F-FDG uptake in a nodule in the left breast. The main differential diagnoses were reactive arthritis, polymyalgia rheumatica, lymphoma or paraneoplastic phenomena. In response to these findings prednisolone 25 mg daily was initiated, which significantly relieved the patient’s symptoms within days. Concurrently, the patient was referred for further evaluation of the PET positive breast nodule, rheumatological evaluation, and a lymph node biopsy was considered.

**Figure 2:**
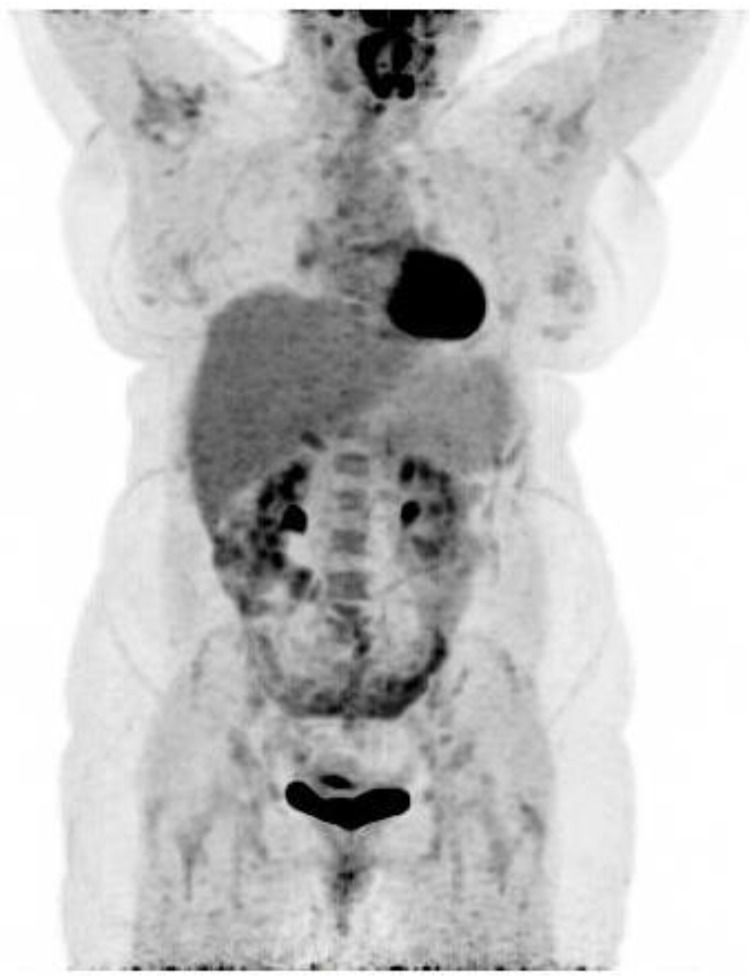
In the meantime, additional diagnostic work-up performed two days prior to the ^18^F-FDG PET/CT, came back with positive Chikungunya IgG and IgM serology. Given the patient’s recent travel to India, the clinical course and positive Chikungunya serology, the patient’s abnormal ^18^F-FDG PET/CT scan was most likely secondary to acute Chikungunya infection and no lymph node biopsy was performed or further evaluations considered. After 12 weeks of gradually decreasing dosage of prednisolone the patient returned to her job as a cleaning lady, but was still reliant on pain-medication. A follow-up ^18^F-FDG PET/CT scan 10 days after ending steroid treatment and 14 weeks after the first ^18^F-FDG PET/CT, showed complete regression of the reactive lymph nodes as well as the ^18^F-FDG uptake in the left breast and only discrete ^18^F-FDG uptake of the right shoulder joint, consistent with the patient’s clinical improvement. Any lingering suspicion of underlying lymphoma was therefore rebuked. A needle biopsy from the left breast showed chronic inflammation but no malignancy. The Chikungunya virus is transmitted by the mosquito vector *Aedes aegypti* and *albopictus* in many parts of the world, including India. Chikungunya viral infection is characterised by flu-like symptoms with high grade fever, headache, myalgia, polyarthralgia (usually symmetrical arthralgia, involving multiple joints) and often accompanied by a maculopapular rash [1]. Severe prolonged arthralgia months to years after infection is a recognised post-infectious manifestation of Chikungunya infection. Symptoms of acute Chikungunya are most likely caused by direct cellular damage and local inflammation with muscles, joints, lymph nodes, skin, liver and spleen as primary sites of replication. Chronic disease seems to be mediated by persistent virus and inflammation [2]. To our knowledge, ^18^F-FDG uptake in a case with confirmed Chikungunya infection has not previously been reported. The ^18^F-FDG PET/CT scan confirmed inflammation of several joints and lymph nodes four weeks after the patient’s initial symptoms. Changes that regressed almost completely. In one report, an Indian patient with suspected Chikungunya infection had ^18^F-FDG uptake in lymph nodes on both sides of the diaphragm including splenic activity and active subcutaneous nodules. However, lymph node biopsy revealed angioimmunoblastic/peripheral T-cell lymphoma [3]. Recently, increased ^18^F-FDG uptake in the spleen and multiple lymph nodes was detected in a patient diagnosed with dengue fever and ^18^F-FDG has been suggested to be a novel Dengue infection-associated inflammation biomarker for assessing treatment response during therapeutic intervention trials [4,5]. ^18^F-FDG PET/CT may be of value in assessing patients suspected for long-term morbidity of Chikungunya infection due to persistent arthralgia.
